# The importance of preoperative T1 slope for determining proper postoperative C2–7 Cobb’s angle in patients undergoing cervical reconstruction

**DOI:** 10.1186/s13018-020-02016-7

**Published:** 2020-11-05

**Authors:** Jinshui Chen, Juying Wang, Xuepeng Wei, Huapeng Guan, Benhai Wang, Hao Xu, Jianmei Chen

**Affiliations:** 1 Department of Orthopedics, 900th Hospital of the Joint Logistics Team, Dongfang Hospital Affiliated to Xiamen University, No. 156 Xi’er huang road, Fuzhou, 350025 China; 2 Department of Nephrology, 900th Hospital of the Joint Logistics Team, Dongfang Hospital Affiliated to Xiamen University, No. 156 Xi’er huang road, Fuzhou, 350025 China

**Keywords:** T1 slope, Postoperative C2–7COBB angle, Cervical reconstruction

## Abstract

**Background:**

This study aimed to explore the relationship among different cervical sagittal parameters in asymptomatic volunteers and the correlation between surgical efficacy and difference of presumed and actual postoperative C2–7 Cobbs’s angle (C2–7COBB), which was calculated based on preoperative T1 slope (T1S) in patients undergoing cervical reconstruction.

**Methods:**

In total, 158 inpatients with cervical spondylosis and 274 asymptomatic volunteers were retrospectively reviewed. Cervical sagittal parameters, such as C2–7COBB, T1S, thoracic inlet angle (TIA), and neck tilt (NT), were compared. Then, the correlation among these parameters was analyzed in asymptomatic volunteers, and a regression equation between T1S and C2–7COBB was established and used to analyze the correlation among the Japanese Orthopaedic Association (JOA) score improvement, the sagittal parameters, and the difference between presumed and actual postoperative C2–7COBB in patients after cervical reconstruction.

**Results:**

The mean T1S, C2–7COBB, and TIA were significantly decreased in patients (*P* < 0.01). T1S and NT had a strong correlation with TIA (*P* < 0.01). T1S demonstrated a moderate correlation with C2–7COBB in asymptomatic volunteers (*r* = 0.569, *P* < 0.01). A regression equation had been established as C2–7COBB = 0.742 × T1S − 0.866. The mean C2–7COBB and JOA score improved significantly (*P* < 0.05) postoperatively. Moreover, the JOA improvement rate showed a significant negative correlation with the difference in the presumed and actual postoperative C2–7COBB (*r* = − 0.696, *P* < 0.01).

**Conclusion:**

Our study successfully established a regression equation for calculating postsurgical C2–7COBB based on the correlation between T1S and C2–7COBB in asymptomatic volunteers. The regression equation could be used for guiding surgeons to accomplish an ideal postsurgical C2–7COBB for patients with cervical spondylosis.

## Background

Cervical spondylosis (CS) is a common degenerative disease. Patients with CS develop facet arthropathy, spinal stenosis, neurologic dysfunction, and pain because the damage to the spinal cord, nerve, and blood vessel is caused by cervical vertebral joint degeneration, hyperplasia, disc herniation, and ligament calcification [1]. Furthermore, these patients develop imbalanced spinal sagittal alignment, which can cause biomechanical instability and worsen the clinical symptoms [2].

At present, cervical sagittal balance-related parameters are gradually used to evaluate the severity and treatment outcomes of the disease. Among the different parameters, the C2–7 Cobb’s angle (C2–7COBB) is commonly used due to its accessibility and high reliability [3]. However, cervical lordosis is influenced by thoracic kyphosis; thus, thoracic inlet alignment such as the thoracic inlet angle (TIA), T1 slope (T1S), and neck tilt (NT) have also been introduced to evaluate the stability of the cervical spine [4, 5]. Among them, T1S is a key factor used to determine the cervical sagittal balance. Thus, a proper relationship between T1S and cervical lordosis might be an important factor for assessing the cervical sagittal balance of asymptomatic volunteers [6].

In people with CS, both T1S and C2–7COBB changes significantly; patients with low T1S (≤ 25) have a higher grade of degeneration than patients with high T1S (> 25) [7]. After cervical reconstruction, the C2–7COBB of the patients changed remarkably, whereas the change in T1S did not show any difference in patients who underwent cervical laminoplasty [8, 9]. Under this circumstance, T1S might be a fixed parameter for patients with CS undergoing reconstruction surgery, and a proper relationship between T1S and other cervical sagittal parameters might result in a better postoperative cervical spine sagittal balance and clinical outcomes. This study aimed to explore the relationship among cervical spine parameters and thoracic inlet parameters among asymptomatic volunteers and patients with CS. Moreover, this study explored the relationship between clinical outcomes and spinal sagittal parameters among patients who underwent cervical reconstruction.

## Methods

### Study design and participants

**Fig. 1:**
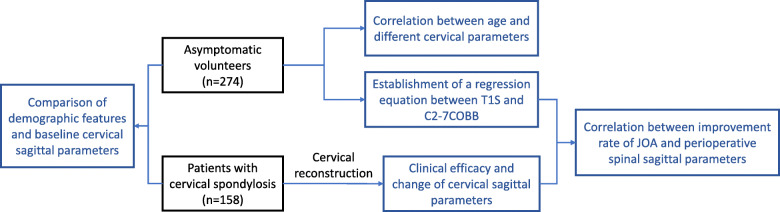
Flowchart of the study

This study enrolled both patients with CS and asymptomatic volunteers for analyzing the correlation among different sagittal parameters of the spine, which included T1S, C2–7COBB, TIA, and NT. Then, a regression equation between T1S and C2–7COBB was established based on asymptomatic volunteers. This equation was used for calculating a presumed postoperative C2–7COBB based on preoperative T1s of patients with CS. The difference between the presumed and actual postoperative C2–7COBB was calculated and then used to explore the correlation between surgical outcome and perioperative spinal sagittal parameters (Fig. 1). This study was approved by the Ethics Committee of Fuzhou General Hospital of Nanjing Military Command, China.

A total of 158 inpatients with CS who received anterior cervical reconstruction treatment at the Orthopedic Department of Fujian 900th Hospital of the Joint Logistics Team from January 2010 to October 2016 were included in the study. The inclusion criteria were (1) aged 40–80 years, (2) the presence of signs and symptoms of spinal cord compression, and (3) imaging findings of multisegment cervical disc herniation and extensive cervical spinal stenosis. The exclusion criteria included (1) a history of cervical spinal surgeries; (2) a history of vertebral destruction, tumor, infection, spinal cord degeneration, muscle atrophy, and abnormalities in the hip, knee, and ankle; and (3) patients with incomplete radiological parameters. Among 158 patients, 96 were male and 62 were female. The average age of the patients was 52.1 ± 9.2 years.

A total of 274 asymptomatic volunteers over 40 years old and without a previously diagnosed spinal disease who came to the hospital for routine physical examination or for diseases that were not related to cervical problems during the same period were enrolled in this study. The mean age was 53.1 ± 9.0 years, and the male-to-female ratio was 159:115.

### Operative management

All patients underwent general anesthesia and anterior cervical corpectomy with fusion or anterior cervical discectomy with fusion for the treatment of CS. The surgeries were performed by experienced physicians. Postoperatively, ECG was monitored for 24 h, and antibiotics and neurotrophic drugs were administered for 48 h. On day 1 postoperatively, patients were asked to sit up with the aid of the collar, and the wound drainage tube was pulled out. In addition, patients also simultaneously started functional exercise using a walking device. Surgical sutures were removed on day 8 postoperatively based on to the healing condition. Furthermore, all the patients wore a cervical collar for 2 months as suggested, and follow-up was done at 3 months postoperatively in all patients.

### Radiological assessment

A professional radiologist conducted lateral radiography of the cervical spine. The patients stood in a comfortable position with the upper limbs falling naturally at each side of the trunk. Moreover, the patients faced forward with a horizontal gaze with the lower jaw slightly raised. Radiological assessment was done for both asymptomatic volunteers and patients with CS. As for patients with CS, a second assessment was conducted at 3 months postoperatively. The values of cervical sagittal parameters were measured twice in each participant, including T1S, C2–7COBB, NT, and TIA. The mean value of each parameter was calculated and used for analysis. C2–7COBB was measured as the angle between the lower endplate of C2 and C7. T1S was measured as the angle between the upper endplate of T1 and a horizontal line. NT was measured as the angle between a vertical line and a line that connected the midpoint of the upper endplate of T1 and the tip of the sternum.

**Fig. 2:**
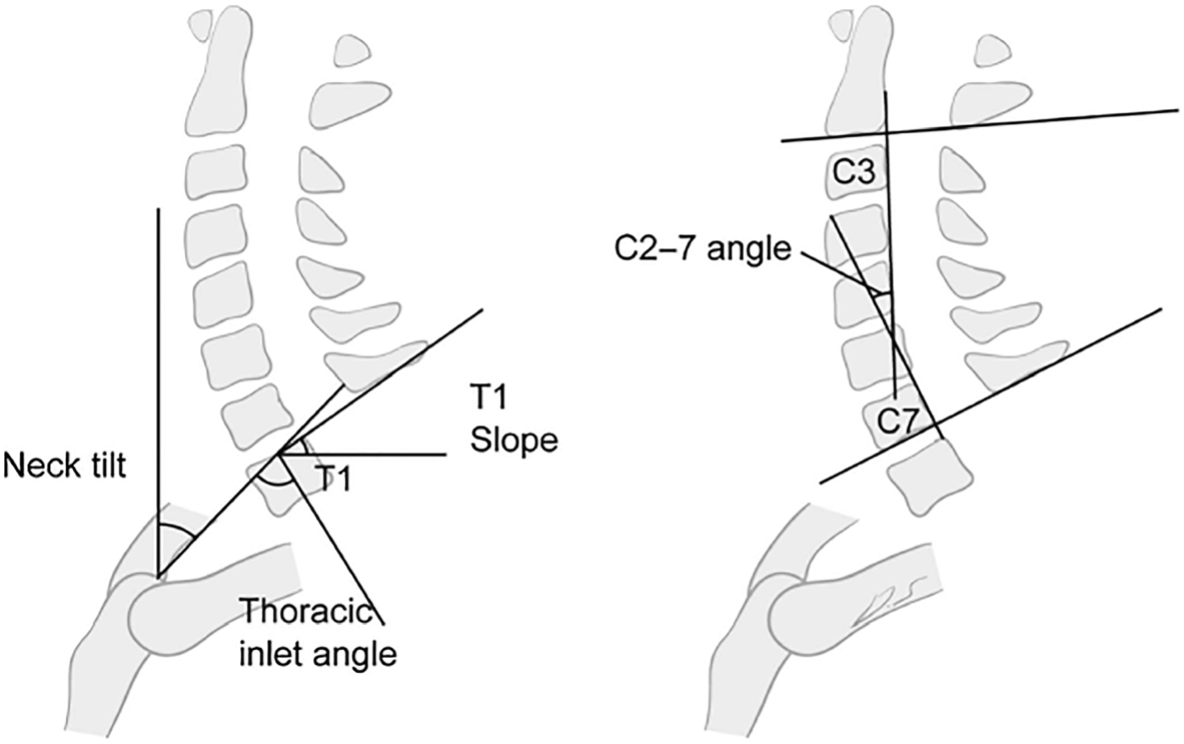
Schematic diagram of cervical sagittal balance parameter measurement

**Fig. 3:**
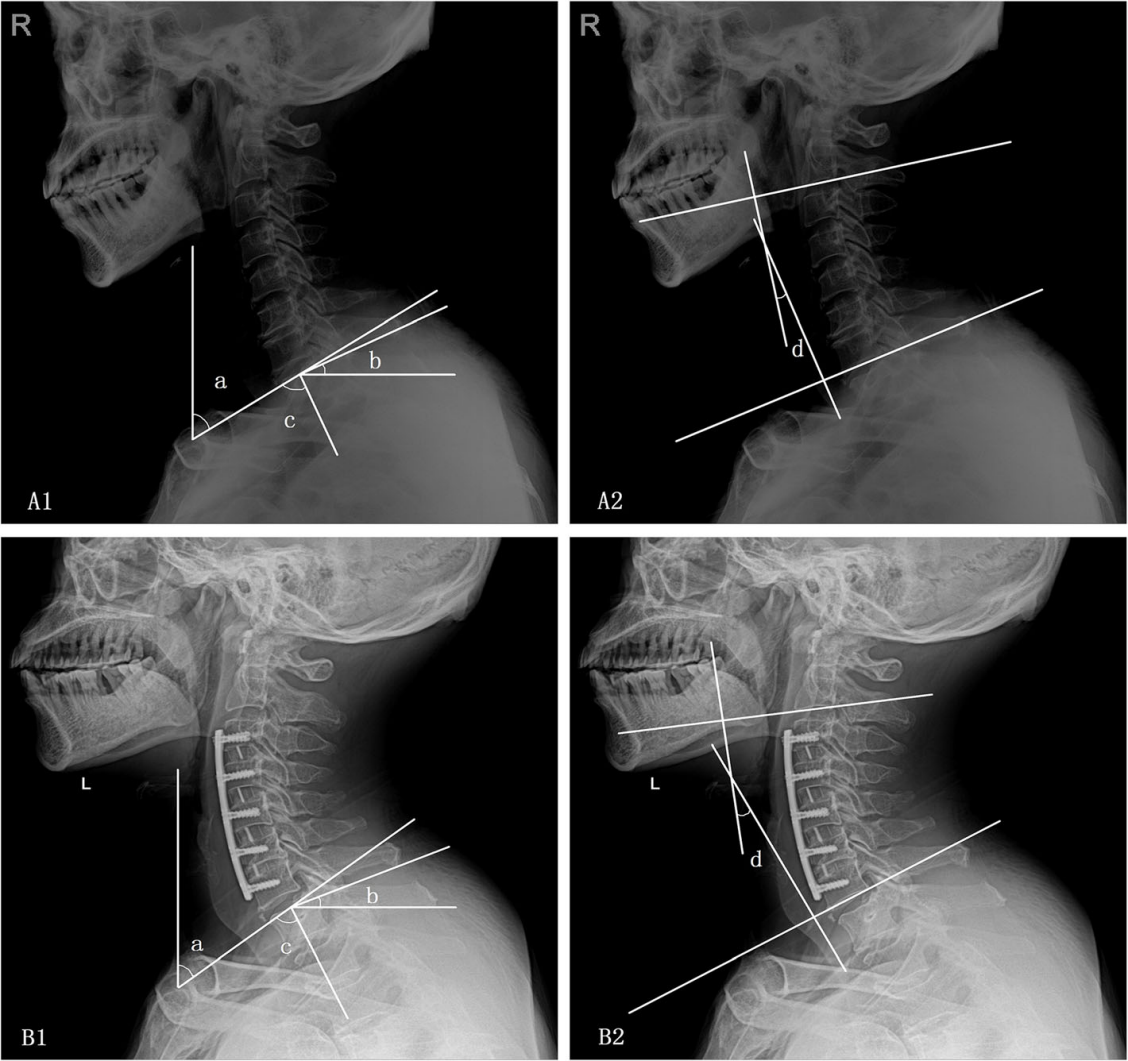
Lateral radiographs of the cervical spine in patients at baseline and at 3 months postoperatively

In addition, TIA was measured as the angle between a vertical line through the midpoint of the upper endplate of the T1 and a line that connected the tip of the sternum and the midpoint of the upper endplate of T1 (Figs. 2 and 3). All the cervical parameters were measured twice by two different evaluators blinded to the study. Different parameters, together with age, were analyzed as a correlation; a regression equation that indicated the relationship between T1S and C2–7COBB was also established.

### Clinical outcome measures

The functional status of patients who underwent surgical treatment was evaluated by the Japanese Orthopaedic Association (JOA) scale at baseline and 3 months postoperatively. The JOA scale indicated the sensory, motor, and bladder functions of the patients. The total score of the JOA scale is 17, with a higher score reflecting a better condition. The improvement rate of the JOA score was calculated using the following formula: [(3-month postsurgical JOA score − preoperative JOA score)/(17 − preoperative JOA score) × 100%] [10]. In addition, this study also recorded the adverse effects affiliated to the reconstruction. Moreover, the former regression equation between T1S and C2–7COBB was applied to calculate the presumed postoperative C2–7COBB. The difference between the presumed and actual postoperative C2–7COBB was calculated and further analyzed for assessing its correlation with the improvement rate of the JOA score.

### Statistical analysis

Statistical analysis was performed using SPSS 18.0 (SPSS Inc., Chicago, IL, USA). Continuous variables are shown as mean ± standard deviation. All continuous variables of patients and asymptomatic volunteers were homogeneous and normally distributed. Student’s *t* test was used to compare patients and asymptomatic volunteers. Paired *t* test was used to compare the baseline and postoperative data in patients who underwent reconstruction. Because age and sagittal parameters were all continuous variables, the Pearson’s correlation coefficient was determined between age, T1S, C2–7COBB, NT, and TIA (0–0.2, no correlation; 0.2–0.4, weak correlation; 0.4–0.6, moderate correlation; 0.6–0.8, strong correlation; and 0.8–1, very strong correlation). Furthermore, the correlation between the improvement rate of the JOA score and other perioperative sagittal parameters was also explored using the Pearson’s correlation analysis. *P* < 0.05 was considered statistically significant.

## Results

Patients with CS had a significantly lower mean T1S (24.0 ± 7.8° vs. 26.4 ± 7.5°, *P* < 0.05), C2–7COBB (14.0 ± 9.7° vs. 18.7 ± 9.8°, *P* < 0.05), and TIA (72.0 ± 9.7° vs. 74.0 ± 10.0°, *P* < 0.05) than asymptomatic volunteers. No significant difference in age, sex, and NT was noted between patients with CS and asymptomatic volunteers (*P* > 0.05; Table 1).

**Table 1:** Demographic features and comparison of the cervical sagittal parameters between asymptomatic volunteers and patients with CS

	Asymptomatic group (*n* = 274)	CS group (*n* = 158)	*P* value
Age, years	53.1 ±9.0	52.1 ±9.2	0.270
Gender, male to female	159:115	96:62	0.612
T1S	26.4 ±7.5°	24.0 ± 7.8°	< 0.05
C2-7COBB	18.7 ± 9.8°	14.0 ± 9.7°	< 0.05
NT	47.6 ±6.6°	47.9 ±6.7°	0.602
TIA	74.0 ± 10.0°	72.0 ± 9.7°	< 0.05

*CS* cervical spondylosis, *T1S-T1* slope, *NT* neck tilt, *TIA* thoracic inlet angle

**Fig. 4:**
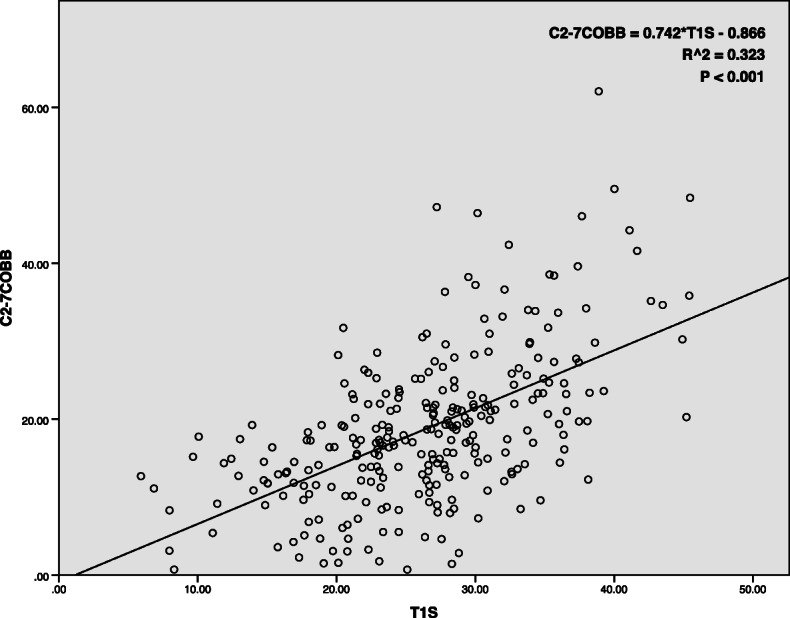
Linear relationship between the C2–7 Cobbs’s angle and T1 slope in asymptomatic volunteers

For the asymptomatic volunteers, T1S (*r* = 0.751, *P* < 0.01) and NT (*r* = 0.666, *P* < 0.01) had a strong correlation with TIA. In addition, a moderate correlation was noted between T1S and C2–7COBB (*r* = 0.569, *P* < 0.01) and between TIA and C2–7COBB (*r* = 0.412, *P* < 0.01). Age showed no relation to other sagittal parameters (Table 2). Based on the highest correlation between T1S and C2–7COBB, a linear regression equation between C2–7COBB and T1S was established as C2–7COBB = 0.742 × T1S − 0.866 (*P* < 0.01; Fig. 4). This regression equation was used to calculate the presumed postoperative C2–7COBB with preoperative T1S.

**Table 2:** Pearson’s coefficients for comparisons of age and different cervical parameters in the asymptomatic group

	Age	T1S	C2-7COBB	NT	TIA
Age	–	0.143*	0.130*	0.104	0.176**
T1S	–	–	0.569**	0.006	0.751**
C2-7COBB	–	–	–	− 0.190	0.412**
NT	–	–	–	–	0.666**

*T1S-T1* slope, *NT* neck tilt, *TIA* thoracic inlet angle

**P < 0.01; *P < 0.05

No severe adverse event was noted in patients who underwent cervical reconstruction. Furthermore, a significant improvement was seen in the mean C2–7COBB (14.0 ± 9.7° vs. 17.3 ± 8.4°, *P* < 0.05) and the JOA score (9.7 ± 1.4 vs. 14.9 ± 1.1, *P* < 0.05) after reconstruction. However, no significant difference was noted among the mean T1S, NT, and TIA at baseline and 3 months postoperatively (*P* < 0.05; Table 3). Moreover, the results showed that the improvement rate of the JOA score had a moderate negative correlation with the difference between presumed and actual postoperative C2–7COBB (*r* = − 0.696, *P* < 0.01), whereas no correlation with other parameters was noted (Table 4).

**Table 3:** Comparison of cervical sagittal parameters between CS patients at baseline and 3 months after surgery

	Baseline (*n* = 158)	3 months after surgery (*n* = 158)	*P* value
T1S	24.0 ±7.8°	24.6 ±6.5°	0.100
C2-7COBB	14.0 ±9.7°	17.3 ±8.4°	< 0.05
NT	47.9 ±6.7°	47.4 ±7.2°	0.183
TIA	72.0 ±9.7°	72.1 ±9.3°	0.795
JOA score	9.7 + 1.4	14.9 + 1.1	< 0.05

*CS* cervical spondylosis, *T1S-T1* slope, *NT* neck tilt, *TIA* thoracic inlet angle

**Table 4:** Pearson’s coefficients between improvement rate of JOA and perioperative spinal sagittal parameters of patients underwent cervical reconstruction

	Improvement rate of JOA, %
Age	− 0.049
Preoperative T1S	0.018
Preoperative C2-7COBB	0.079
Preoperative NT	0.057
Preoperative TIA	0.053
Postoperative T1S	− 0.116
Postoperative C2-7COBB	− 0.027
Postoperative NT	− 0.062
Postoperative TIA	− 0.129
Presumed postoperative C2-7COBB	− 0.116
Difference between presumed and actual postoperative C2-7COBB	− 0.696**

*T1S-T1* slope, *NT* neck tilt, *TIA* thoracic inlet angle

**P < 0.01

## Discussion

In this study, we compared the cervical sagittal parameters between asymptomatic volunteers and patients with CS. The correlation among the different parameters was also analyzed in asymptomatic volunteers, and a regression equation between T1S and C2–7COBB was established to calculate the presumed postoperative C2–7COBB in patients undergoing cervical reconstruction. Furthermore, for patients who underwent reconstruction, the relationship between the improvement of JOA score and the perioperative sagittal parameters was also explored.

We found that the patients with CS had a lower T1S and C2–7COBB than the asymptomatic volunteers, which was in accordance with the study by Yang et al. [7] who reported that patients with cervical degeneration presented with low C2–7COBB and T1S. Moreover, C2–7COBB had a positive correlation with T1S. Stress concentration develops if the cervical lordosis becomes straightened, which later causes CS [11]. As T1S decreases, a higher stress concentration is added onto the lower cervical spinal segments, such as C5–6 and C6–7, which worsens the symptoms of CS in the end and accelerates the process of degeneration. However, Ma et al. [12] conducted a study that compared T1S between patients with and without Modic changes (MCs). The results showed that patients with MCs had a significantly higher T1S than those without MCs. In this study, only patients with symptoms were enrolled. The patients without MC had a mean T1S of 20.6°, which contradicted the result of another study which indicated that the mean T1S in the asymptomatic adult volunteers was 25.7° [5]. In this situation, we believed that the criteria of the participants might be the key factor that induced the difference.

As for the correlation among the different sagittal parameters, our study showed that T1S and NT had a strong correlation with TIA. Lee et al. reported that TIA was equal to T1S plus NT, and TIA had a significant moderate correlation with T1S [5]. In another study, Weng et al. also found that TIA had a moderate correlation with T1S, whereas TIA had a strong correlation with NT [13]. C2–7COBB is a sensitive parameter for the evaluation of postoperative cervical lordosis correction [14]. In our study, we found that C2–7COBB had a moderate correlation with T1S and TIA, which was in accordance with the results of other studies. Wang et al. reported that C2–7COBB had a moderate correlation with T1S and TIA in asymptomatic volunteers, and a weak correlation existed between these parameters in outpatients [15]. Zhang et al. also found that C2–7COBB had a moderate correlation with T1S [16]. It was assumed that people should have a large C2–7COBB to perform a horizontal gaze while they had a high T1S. The imbalance between C2–7COBB and T1S might be a key factor that accelerated the progression of degeneration [12, 17].

At present, there is a conflict between the improvement of neurological function and restoration of the physical curvature of the cervical spine. Suda et al. [18] reported that the signal intensity changes in MRI and local kyphosis were the most important risk factors for poor surgical outcomes. Kawakami et al. [19] believed that postoperative reconstruction of the cervical curvature was the key to improving neurological symptoms in patients with degenerative changes. Hirabayashi et al. also supported that the correction of spinal alignment was important for achieving good surgical outcomes of expansive laminoplasty [20]. However, in other studies, Grob et al. showed that there was no association between any of the clinical features (such as duration, intensity of pain, and disability) and cervical curvature in volunteers over 45 years old [21]. Hojo et al. found that excessive kyphosis correction could increase the risk of complications such as foraminal stenosis in patients who underwent cervical reconstruction [22]. In our study, we found that the C2–7COBB changed significantly postoperatively, and the JOA score was also significantly improved. Postoperatively, T1S and TIA did not change remarkably, which was in accordance with the study by Cho et al. [9] who found a significant change in C2–7COBB but no change in T1S in patients’ postcervical laminoplasty. Moreover, Gillis et al. found that aside from C2–7COBB, T1S showed no significant change after the patients received anterior cervical discectomy and fusion, which indicated that T1S might be a fixed parameter to predict the ideal postoperative C2–7COBB [23]. In our study, we adopted a regression model between T1S and C2–7COBB to calculate the presumed postoperative C2–7COBB, and then, the difference between the presumed and actual postoperative C2–7COBB was calculated and analyzed. In addition, we found a significant negative correlation between the difference of C2–7COBB and the improvement rate of the JOA score. Under this circumstance, we believed that the restoration of cervical curvature was beneficial for clinical outcomes and that it is possible to use a fixed T1S to predict the presumed C2–7COBB after reconstruction and help the surgeon set the goal for reconstruction.

This study had some limitations. First, this study was a single-center study, and the number of participants was relatively small. Second, the follow-up evaluation of this study was only at 3 months, and there was still a possibility of changes in these parameters in a longer follow-up period. Our study first introduced the regression model between T1S and C2–7COBB, which might be a proper and useful method for predicting the presumed postsurgical C2–7COBB and surgical goal setting. In the future, larger scale studies should be conducted to verify the use of this regression model in the management of patients with CS.

## Conclusion

T1S, C2–7COBB, and TIA were significantly decreased in patients with CS. For asymptomatic volunteers, T1S had a significant correlation with C2–7COBB. A regression equation was established for calculating postsurgical C2–7COBB based on the correlation between T1S and C2–7COBB in asymptomatic volunteers. In patients with CS who underwent reconstruction, the JOA score was improved significantly. The deviation of C2–7COBB between the predicted C2–7COBB and actual C2–7COBB might be a risk factor for JOA remission. The regression model might be used in the figure for guiding surgeons to set an ideal postsurgical C2–7COBB for patients with CS.

## Data Availability

All data generated or analyzed during this study are included in this published article.

## Abbreviations

*T1S*: T1 slope
*TIA*: Thoracic inlet angle
*NT*: Neck tilt
*JOA*: Japanese Orthopaedic Association
*CS*: Cervical spondylosis
*MC*: Modic change
